# The current and potential uses of Electronic Medical Record (EMR) data for primary health care performance measurement in the Canadian context: a qualitative analysis

**DOI:** 10.1186/s12913-021-06851-0

**Published:** 2021-08-15

**Authors:** Erica Barbazza, Sara Allin, Mary Byrnes, Andrea D Foebel, Tanya Khan, Patricia Sidhom, Niek S Klazinga, Dionne S Kringos

**Affiliations:** 1 Department of Public and Occupational Health, Amsterdam UMC, University of Amsterdam, Amsterdam Public Health research institute, Meibergdreef 9, 1105 AZ Amsterdam, the Netherlands; 2 Institute of Health Policy, Management & Evaluation, University of Toronto, Toronto, Canada; 3 Canadian Institute for Health Information (CIHI), Toronto, Canada

**Keywords:** Performance indicators, Electronic Medical Records, Quality of health care, Canada, Primary health care, Quality measurement

## Abstract

**Background:**

Electronic Medical Records (EMRs) are a rich data source to measure and improve quality of care. As Canadian primary health care (PHC) EMRs mature, there is increasing potential use of EMR data for performance measurement. This study identifies and describes current uses of EMR data for performance measurement and considerations to further its potential in the Canadian context.

**Methods:**

We applied a qualitative case study design and descriptive assessment in three phases, consulting multiple data sources including scientific and grey literature, system leaders (*n* = 41), and clinician/researchers (*n* = 20). Phases included a multimethod approach to identify initiatives using EMR data for performance measurement across Canadian jurisdictions; in-depth review of current initiatives identified from a healthcare performance intelligence lens; and triangulation and thematic analysis across data sources to explore considerations for advancing performance measurement uses of EMR data in the Canadian context.

**Results:**

Six initiatives of EMR data use for performance measurement were identified: one multi-jurisdictional; five jurisdiction-specific in the provinces of British Columbia, Manitoba and Ontario. EMR data uses were predominately for micro-level PHC physician and team performance improvement, with some use for meso-level organization/network-wide improvement. Indicator sets varied in number, though shared emphasis on chronic disease management and prevention/screening and to a lesser extent medication management. Key considerations for governing, resourcing and implementing EMR data for performance measurement were identified.

**Conclusions:**

The extent of EMR data use for performance measurement varies across Canada. To further its potential, pan-Canadian data and privacy standards, performance intelligence competencies and renewed core PHC indicators should be prioritized. Experiences across countries, coupled with increasing momentum for performance measurement using real-world data, should be leveraged to avoid unnecessarily slow progress in Canada and abroad.

**Supplementary Information:**

The online version contains supplementary material available at 10.1186/s12913-021-06851-0.

## Background

The evidence base for primary health care (PHC) as an accelerator towards universal health coverage and enhanced population health has sustained a PHC approach to services delivery as the ambition of countries worldwide for decades [1–5]. Measuring the performance of health services has a fundamental role to play in assuring quality of care and achieving improvements [6]. By definition, performance measurement “seeks to monitor, evaluate and communicate the extent to which various aspects of the health system meet key objectives” [7]. The resulting performance intelligence has important uses that extend across the micro-meso-macro contexts of health systems. These uses include, for example, improving the management of a practice panel by individual physicians or PHC teams at the micro-level, assuring care standards are adhered to across networks or community health centres at the meso-level, or identifying gaps in care for population subgroups to inform strategic priorities at the macro-level [6, 8, 9].

Electronic medical records (EMRs) are an important data source for clinical care but also for secondary uses, including performance measurement. The rich patient-level data generated in EMRs has a number of advantages relative to other PHC data sources, such as administrative data or surveys. This includes its granularity, especially for diagnosis and intervention-related information [10, 11], and its potential to link with other data sets, such as hospital discharge data. The timeliness of EMR data is also a key advantage, with increasing potential for near–real-time data extraction. The value of its timeliness has been demonstrated in the context of the COVID-19 pandemic. For example, countries with advanced secondary uses of EMR data, such as the Netherlands [12] and United Kingdom [13], have leveraged EMRs as a source for measuring the spread of community infection and its impact on population health and health services.

Despite the advantages of EMR data, realizing its full potential for performance measurement uses across health systems faces a number of challenges. This includes the quality and utility of its hybrid structured, semi-structured and unstructured data [8, 14]. Other challenges across countries have traditionally included the low penetration of EMRs, insufficient analytical capacity to make use of the data, and inconsistent use of minimum or standard data elements [14, 15].

In the Canadian context, each of the 13 provinces/territories have followed their own approach to EMR implementation since the early 2000 s [16]. The differing paths taken and level of prioritization for EMR content standards, have resulted in varied EMR systems across the country. The ensuing patchwork of EMRs [16], persistent variability in EMR adoption rates, and ultimately, limitations in data quality and comparability, have each in part contributed to the slowed use of EMR data for performance measurement [17–19].

Nonetheless, the PHC EMR environment in Canada is changing. In 2019, 86 % on average of participating Canadian family physicians to the Commonwealth Fund International Health Policy survey reported using EMRs in their practice [20]. This figure, while still below the Commonwealth average (93 %) [20], has more than doubled in the past decade, up from 37 % to 2009 [21], and 73 % in 2015 [22]. The development of pan-Canadian EMR content standards and minimum data set [23, 24], and assessments of EMR benefits [25], are signs of continued progress and sustained momentum [19]. As the adoption and sophistication of EMR systems advances, the lament of limited, quality EMR data has been described as a deficit that has continued to shrink [26, 27].

In this study, we set out to systematically identify and describe the current uses of EMR data for performance measurement in Canada. We additionally aimed to explore challenges to be overcome for furthering the potential uses of EMR data for PHC performance measurement. To do so, we explored the following three questions in the Canadian context: Where is EMR data currently used as a source for performance measurement? What are the purposes of use and indicators sourced from EMR data for the initiatives identified? And, what are key considerations to furthering the use of EMR data for PHC performance measurement?

## Methods

### Design

**Fig. 1:**
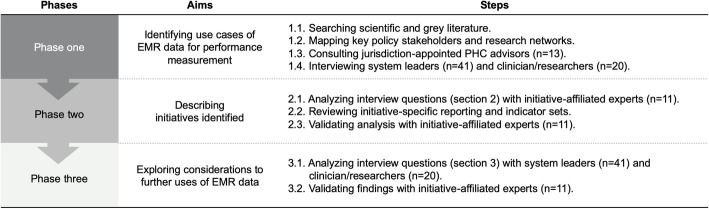
Overview of study phases

We employed a qualitative case study design and descriptive assessment in three phases (Fig. 1) [28]. Reporting is in accordance with the Standards for Reporting Qualitative Research [29]. First, we consulted multiple data sources, including system leaders and researcher/clinicians across Canadian jurisdictions, to systematically identify use cases (initiatives) of EMR data for performance measurement. Second, where identified, these initiatives were studied in-depth from a health care performance intelligence lens according to an existing characterization of *fit for purpose* and *fit for use* healthcare performance indicators [9]. Third, to explore the further potential uses of EMR data in the Canadian context, we triangulated and analyzed data collected in a deductive and inductive approach using thematic analysis [30–32].

The first author is a doctoral student in healthcare performance intelligence focusing on the actionability of healthcare performance data. The multidisciplinary study team consisted of experts with complementary research, policy and subject matter expertise in the Canadian context.

We defined initiatives of EMR data for performance measurement as established processes to extract, analyze, and display (report) EMR data for quality of care-related decision-making [7, 9]. No restrictions were placed on the primary decision-making context (e.g. micro-, meso-, macro-level uses) or type of organization responsible for the initiative’s development (e.g. government agency, professional association, research network). In line with our aim to describe the context and processes of initiatives in practice, we excluded initiatives in the initial stages of development (pre-implementation), though included initiatives that had ended within the past year.

### Setting

In Canada, the 13 provincial and territorial governments steward PHC services for their populations [33]. This autonomy accounts in part for the variation across jurisdictions with regards to how a PHC approach is defined, including its delivery as primary care services, organization of practices (e.g. solo physician, group or multi-profile practices) [20] and payment of providers (e.g. fee-for-service, salaried, capitation, blended models) [34]. There is similar heterogeneity in how jurisdictions approach performance measurement and improvement: some with dedicated agencies (quality councils), and others assigning this role to a ministry department or regional health authority and/or professional associations [35–37]. These differences also extend to measurement itself, with jurisdiction-defined performance frameworks and indicator sets.

To facilitate jurisdiction-led PHC performance measurement and to encourage meaningful comparisons within and across jurisdictions, a core set of pan-Canadian PHC indicators was first developed in 2006 and updated in 2012 [38, 39]. At the outset, administrative and survey data were suggested data sources. In the 2012 update, the primary care EMR system was added as a possible source for a subset of indicators. The use and sources of these indicators is ultimately to the discretion of each jurisdiction.

### Phase one: identifying use cases of EMR data for performance measurement

In the absence of an up-to-date overview of EMR data as a source for performance measurement in Canada, we first explored uses and sources of PHC performance measurement across jurisdictions. We took as a basis a related environmental scan conducted by the Canadian Institute for Health Information (CIHI) (CIHI, unpublished data, 2016). From this, an initial listing of initiatives was developed. We used multiple methods to systematically update this list.

First, the scientific and grey literature on PHC performance measurement in Canada was searched. Searches were conducted using PubMed in late-2019 using the following key terms in varied combinations: EMR; performance measurement; PHC; Canada. Reference lists of relevant literature were reviewed in a snowballing approach.

Second, we identified and mapped more than 80 key policy stakeholders and research networks related to PHC performance measurement and/or improvement by jurisdiction (Supplementary file 1). Websites of identified organizations were searched manually for relevant reporting or activities. French-language websites were reviewed using online translations.

Third, an existing CIHI network of jurisdiction-appointed PHC advisors (*n* = 13)—comprising executives in roles related to PHC from provincial/territorial ministries of health—was convened virtually in February 2020 to validate the completeness of the actors and mapping of initiatives, and to solicit insights on other emerging efforts. Recommendations for jurisdiction-specific experts to consult were also sought. All comments and discussion points were documented, and members were followed-up with by email.

Lastly, we directly consulted with experts across jurisdictions for their firsthand insights into their respective contexts. Two profiles of experts in each jurisdiction were pursued: (i) system leaders affiliated to provincial/territorial ministries of health, health authorities, quality councils, professional associations and/or other key stakeholders; and (ii) researchers affiliated to academia, research networks and/or practicing clinicians. The large number of experts was deemed necessary given the exploratory aims of the study.

Individuals were identified by drawing on contact and membership lists of webpages consulted, authorship of literature reviewed, expertise of the study team and advisors met with, as well as a snowballing of recommendations. We contacted 91 experts via email in English or French, providing an overview of the study and in total, 61 experts were consulted: 41 system leaders and 20 clinician/researchers. See Supplementary file 3 for an overview of experts by jurisdiction. We requested to engage each in one-on-one discussions, rather than written responses, for rich individual exchanges and practical insights into reasons contributing to contexts where EMR data was not leveraged as a source for performance measurement (research question 3). See Supplementary file 2 [40] for further details on the topics and approach taken.

Data was collected over a three-month period (January–March 2020). This phase was considered complete when at least one of the target two profiles of experts was consulted from each jurisdiction. Non-participants (*n* = 30) were nearly equal-thirds unreachable, unavailable or referred to an alternate contact. The target two perspectives (system leaders and clinician/researchers) were met in 8 of 13 jurisdictions. In one instance (Yukon), researchers working elsewhere but with experience working in the jurisdiction were consulted in lieu of available informants. All discussions took place in English and were conducted by the first author joined by one team member (SA, MB, TK), primarily for consultations conducted with two or more informants. In three instances, information was collected via email exchanges only.

### Phase two: describing initiatives identified

**Fig. 2:**
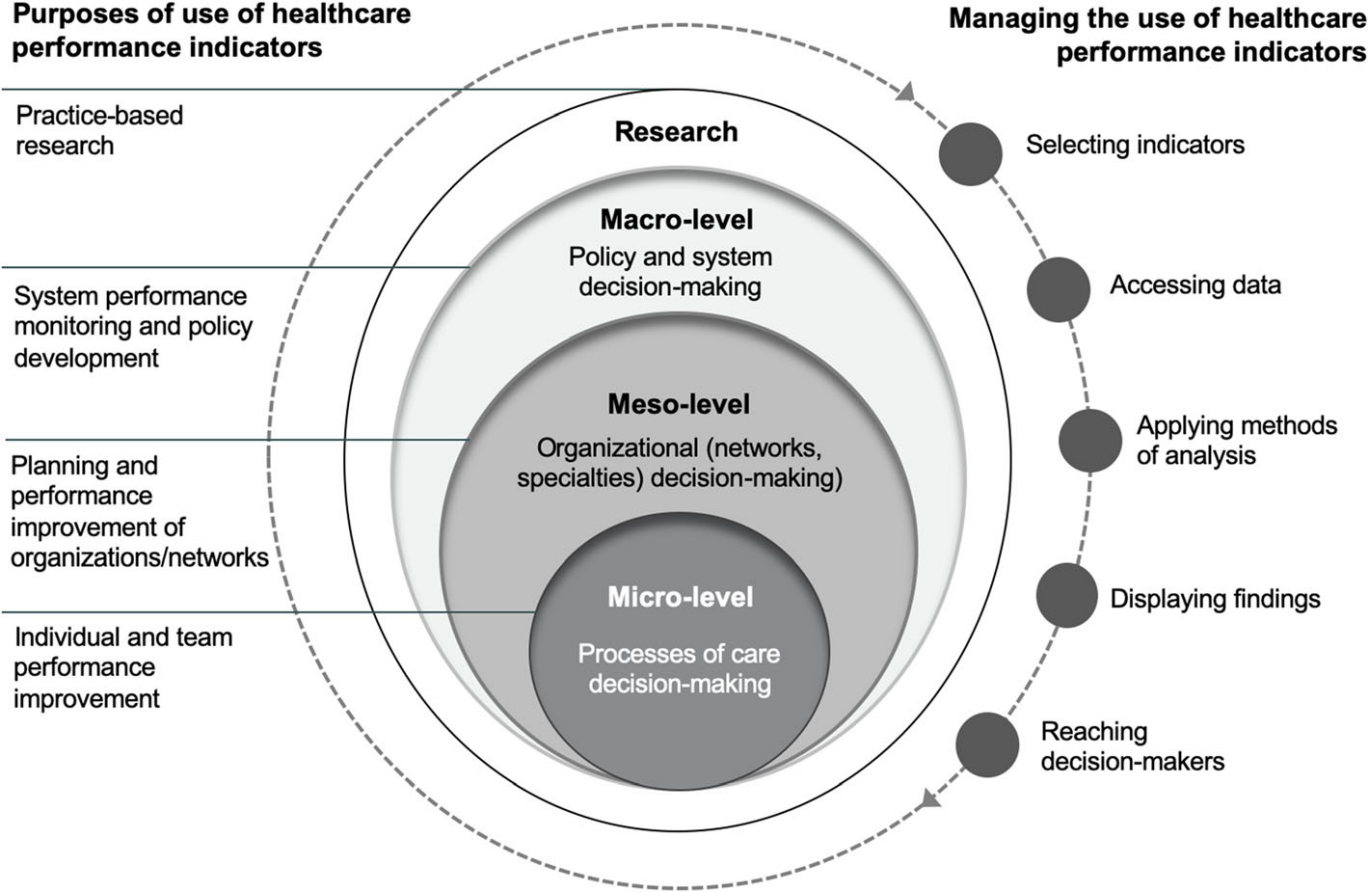
Conceptualization of purposes of use and fitness for use considerations applied to describe initiatives

We developed a description of the initiatives identified from a healthcare performance intelligence perspective in the approach visualized in Fig. 2. Creating healthcare performance intelligence accounts for the varied steps to convert data to indicators, information to knowledge, and use of this knowledge as action in decision-making [40]. To be actionable, data should be both fit for purpose and use [9]. We applied these constructs to describe each initiative by their intended purpose and management in practice.

Specifically, we differentiated the *uses* of EMR data for performance measurement beyond their common aim of informing quality of care-related decision-making. These *uses* were distinguished according to an existing multi-level characterization as improving individual or team performance (micro-level); planning and performance improvement of organizations/networks (meso-level); system performance monitoring and policy development (macro-level); and cross-cutting uses for practice-based research [9, 41]. To depict the handling of data in practice, we applied the conceptualization of an indicator’s use cycle, extending from the selection of indicators to processes for accessing the data, analyzing and displaying results and reaching the target decision-makers.

We used multiple data sources to describe the initiatives identified. This included supplementary questions during semi-structured interviews with the aforementioned experts directly involved in these initiatives (Supplementary file 2, Sec. 2). Records of the interviews were prepared as detailed summaries. We triangulated data sources to prepare a description of the cases in the approach described (Fig. 2). The experts consulted from each initiative were returned the analyzed findings to review its completion and accuracy. Two follow-up discussions were organized and other written feedback was incorporated into the description of each case.

### Phase three: exploring considerations to further uses of EMR data

As a final phase, we explored the underlying main challenges to further the use of EMR data as a source for performance measurement in the Canadian context. Thematic analysis was used to analyze the collected data of the first two phases [32]. Data analysis was performed manually in a deductive and inductive approach [31, 32]. The deductive analysis was guided by the considerations explored related to the management of healthcare performance indicators (selecting indicators, accessing data, applying methods of analysis, displaying findings and reaching decision-makers) [9]. We also applied the main categories of contextual considerations previously found to influence an indicator’s use defined as information infrastructure, governance, workforce capacity, and culture [9]. Additional themes and naming subcategories emerged in an inductive approach. The initial coding and clustering of themes was conducted by the first author and reviewed by the study team.

A preliminary analysis of the study findings was presented at a public webinar in April 2020. All experts contributing to the earlier phases of the study were personally invited to attend the event. The event was attended by approximately 100 participants. As such, the presentation of preliminary findings gave an opportunity for member checking. The final clustering of main challenges was also reviewed by the experts of the six initiatives consulted to review the results of phase two.

### Ethics

The research adheres to the Dutch ethics guidelines stated in “Medical Research Act with People (Wet medisch-wetenschappelijk onderzoek met mensen (WMO)) [Dutch], in BWBR0009408, W.a.S. Ministry of Health, Editor. 1998: Hague, Netherlands” [42], for which verbal consent was deemed adequate by the authors as no human data was retained. To ensure informed voluntary participation, experts contributing to this study provided written agreement to participate during the recruitment stage.

## Results

### Identified initiatives of PHC performance measurement using EMR data

Across the jurisdictions, we identified six initiatives—one multi-jurisdictional and five jurisdiction-specific (British Columbia, Manitoba, Ontario)—where EMR data is used as a source for measuring PHC performance. Table 1 describes the six initiatives. The actors underpinning each vary, ranging from ministries of health, to membership-based networks, to actors with a mandate focused on EMR data use. Funding is predominately from the respective ministries or grant-specific. Importantly, the underpinning payment model for affiliated practices varies across initiatives, ranging fee-for-service, capitated and salaried practices. The initiatives range from well-established, having been in place for more than five years, to more recent like *Health Data Coalition’s (HDC) Discover* and *OntarioMD’s Insights4Care*. In June 2020, all initiatives were being implemented aside from Association of Family Health Teams of Ontario’s (*AFHTO) Data2Decisions (D2D)* which was time-bound and ran between 2014 and 2019, though its resources remain in the public domain [27].

Further to these established initiatives, a number of pilot or emerging examples of EMR data use were identified. These include: the Quebec-based initiative *Le Collectif pour les Meilleures Pratiques et l’Amélioration des Soins et Services+* (CoMPAS+, the Collective for Best practices and Improvement of Care and Services) exploring EMR data as a source for practice feedback [43, 44]; the *Community Information Integration* initiative in Alberta, working to centrally store EMR data for quality improvement and system planning [45]; and in Saskatchewan, the *Chronic Disease Management Quality Improvement Program* using EMR data together with paper-based records for issuing quality improvement payments [46]. We also identified a number of research-focused initiatives including the multi-jurisdictional project *SPIDER* [47], and Quebec-based initiative *PULSAR* [48]. The experts also described a number of ad hoc, physician-driven initiatives that have emerged organically as physicians champion the use of their EMRs (e.g. [49]).

### Description of six initiatives

For the purpose of this study, the six initiatives of EMR data for performance measurement were explored further (Table 1). Overall, measurement was found geared towards the micro-level context to improve the performance of individual physicians or teams. Two initiatives, *D2D* and the *Business Intelligence Reporting Tool (BIRT)* additionally target the meso-level context, using EMR-sourced indicators for planning and improvement of community health centres and family health teams/organizations. Similarly, *HDC Discover* and *Insights4Care* are also expanding to meso-level uses for communities and integrated health teams, respectively. No macro-level uses of EMR data were identified.

Each initiative has developed processes to extract, anonymize and centrally-store EMR data for affiliated practices, with the exception of *Insights4Care* which queries data directly from patient files. The frequency and automatization of data extraction processes vary, with more manual efforts in some instances, such as *D2D*’s approach requiring data uploading to a secure platform on a 6-month cycle. This is in contrast to *BIRT* and *Insights4Care* which extract data from the EMRs daily.

With regards to the analysis of data, the initiatives were found to share a common approach to report indicators over time and using breakdowns that range for comparisons between practices, organizations and/or the province. The initiatives vary in the frequency of data updating, from daily, to quarterly to every 6-months. In all instances, the detailed analyzed data is not publicly reported and rather, is presented in secure online dashboards or portals, aside from in Manitoba where feedback is provided as offline reports. Informants across the initiatives emphasized the support of hands-on data quality improvement specialists, though the approach and availability of such resources ranged from at-distance (e.g. in Manitoba), to partnership-driven (e.g. *HDC Discover*), to practice-affiliated data and improvement specialists (e.g. *D2D, BIRT, Insights4Care*).

Some user feedback and evaluations on the impact of initiatives have been conducted, like in the case of the *Canadian Primary Care Sentinel Surveillance Network (CPCSSN)* and its *Data Presentation Tool* [50–52], the pilot phase of *Insights4Care* [53], and *AFHTO’s D2D* [54–56]. Assessing the impact of each initiative was beyond the scope and aims of this study.

**Table 1:** Overview of EMR data for PHC performance measurement by initiative

Considerations	CPCSSN	HDC Discover	Manitoba PCQI	Insights4Care	D2D	BIRT
**Context**						
Jurisdiction	Multiple	British Columbia	Manitoba	Ontario	Ontario	Ontario
Actor	CPCSSN	Health Data Coalition	Manitoba Health	OntarioMD	AFHTO	The Alliance
Funder	Public Health Agency of Canada	General Practice Services Committee	Manitoba Health	Ontario Ministry of Health	Ontario Ministry of Health	Multiple sources; not-for-profit
Duration of initiative	> 5 years	< 5 years	> 5 years	< 5 years	5 years (ended 2019)	> 5 years
**Purposes of use**						
Target context	Cross-cutting	Micro	Micro	Micro	Micro/Meso	Micro/Meso
Primary use	Practice-based research	Individual performance improvement; community improvement	Team performance improvement	Individual performance improvement	Planning and improvement of FHT/team	Planning and improvement of CHC/team
Target users (practice type)	Individual physicians; practice-based researchers	Individual physicians/ NPs (solo/group practices)	Home clinic teams (group practices)	Individual physicians, NPs and practice staff (solo/group practices)	Individual physicians, teams, executives (FHTs)	Individual physician/NPs, executives (CHCs)
**Managerial considerations**						
Number of indicators	NA^a^	184	44	64	17	40+
EMR vendors	Spans across multiple vendors	OSCAR, MOIS, Telus Health, Intrahealth	Spans across multiple vendors	Telus Health, OSCAR	Across yet mainly: Telus Health, Accuro, OSCAR	Telus Health, NOD, Purkinje
Analysis frequency	6-month	Quarterly	Quarterly	Daily	6-month	Daily
Feedback format	Portal dashboard	Portal dashboard	Report	EMR-based dashboard	Portal dashboard	Dashboard and report
Public reporting	No	No	No	No	Yes (summaries)	Yes (annual report)
User support	Local-network led	Collaboration with practice support program	At-distance supportof department at Manitoba Health	Practice Enhancement Consultants	Network of QIDSS	CHC-based data coordinators; region-al decision support
Evaluations of initiative	Multiple studies; user feedback	User feedback	Ad hoc	Proof of concept evaluation	Project evaluation	Ad hoc

^a^ As a surveillance database, varied data elements are collected and can be reported on by CPCSSN

**Fig. 3:**
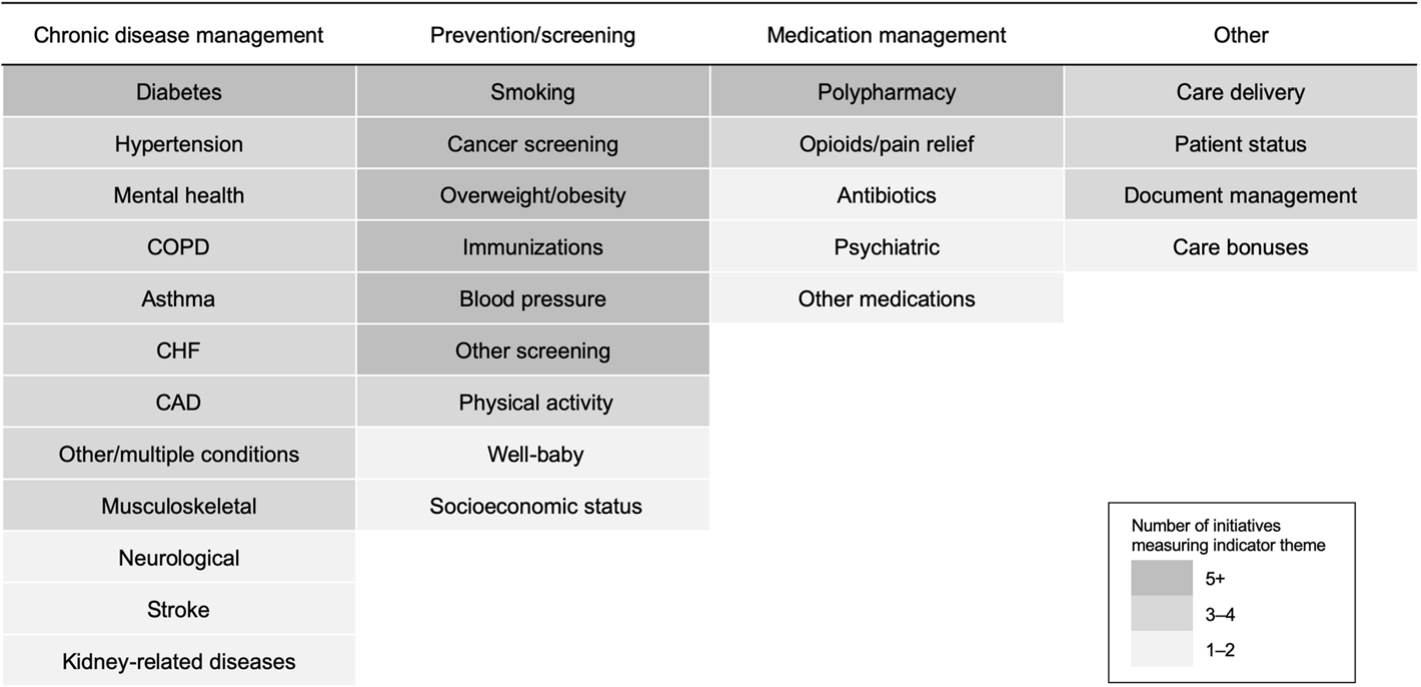
Common indicator themes across EMR-sourced indicators by initiative. *COPD* chronic obstructive pulmonary disease, *CHF* congestive heart failure, *CAD* coronary artery disease

We explored the common themes—as the focus of indicators—being measured across the initiatives. Figure 3 summarizes recurrent themes in four main clusters: chronic disease management, prevention/screening, medication management and other measures. See Supplementary file 4 for a detailed mapping of the frequency of themes by initiative. The most common themes were related to prevention/screening including smoking, cancer screenings, obesity, immunizations and blood pressure. Screening by socioeconomic risk factors, such as food and housing insecurity, was uniquely captured by one initiative (*BIRT*). EMR data was frequently used by the initiatives to measure chronic disease management, in particular diabetes as well as cardiovascular diseases, mental health and respiratory diseases. Measurement related to prescribing was less common beyond polypharmacy patients. Indicators related to care delivery, such as follow-up after hospitalization, hospital admissions for ambulatory care sensitive conditions or emergency department visits, were reported with medium frequency.

### Key considerations to extend EMR data use

Canadian jurisdictions are at varied stages of development to use their EMRs, from early EMR adoption to improving and extending its use like in the initiatives identified. Despite these differences, our analysis across data sources and jurisdictions found commonalities in challenges to further the use of EMR data. Specific challenges emerged related to governance, contextual and implementation fitness for use considerations (Table 2). The identified initiatives, while few in total, offer some local solutions based on the experiences of these efforts to-date. For example, among the main contextual challenges are those related to the time and resources demanded to improve the quality of data due to lack of common regulations and data standards. The initiatives studied offer different approaches to address this, from increased attention and prioritization of data standards to hands-on support in-practice.

**Table 2:** Summary of common considerations for increasing EMR data use for performance measurement in the Canadian context

Consideration	Main challenge	Lessons from initiatives
**Governance**		
Vision and political will	Gaining momentum to establish privacy and technology regulations and prioritize use of data due to lack of high-level commitment.	Build indicators into new PT-initiatives, strategies or reforms; define clear roles and uses of data from the outset.
Privacy and data sharing regulations	Clarifying the relationship between patients, physicians and vendors regarding data ownership versus custodianship.	Engage across stakeholders from the outset including data users; improve utilisation of existing standards.
Aligned financing structures	Ensuring PHC workforce will be paid for their time due to different payment models in primary care.	Embed measurement and improvement into payment system for fee-for-service PHC physicians; consider incentives (financial and non-financial) for salaried physicians.
**Contextual**		
Information system infrastructure	Lagging saturation of EMRs due to time and resource burden of negotiating with vendors and standardizing the information architecture.	Leverage developed tools from vendors for use in other contexts to accelerate progress; prioritize standardization from the outset.
Data quality	Investing considerable time and resources to improve the quality of data due to lack of common regulations specifying data standards.	Standardize what, how and where information is to be recorded in patient records; increase use and adherence to standards through trainings.
Workforce capacity	Ensuring PHC professionals appreciate the importance for high quality data capture and its use due to lack of training in population health and quality improvement.	Define and invest in data literacy as a PHC professional competency; ensure all levels are equipped with performance intelligence competencies.
Professional culture	Changing behavior and professional culture due to misaligned accountability, concerns of trust, time span needed for behavior change and critical mass of users.	Engage champions to demonstrate data use in practice; integrate data use into accountability arrangements.
**Implementation**		
Selecting indicators	Selecting meaningful indicators due to unclear purposes of use, undefined priority indicators, challenges to capture multi-professional teamwork.	Ensure the intended use of data is clear from the outset; standardize core indicators; continuously review indicator sets with end-users.
Accessing data	Configuring across EMR vendors to gain access to data due to varied vendors with unharmonized standards and lack of regulations for EMR vendors.	Standardize workflows for data entry; support PHC professionals through initial and continuous training.
Displaying findings	Designing a simple, user-friendly display of findings due to differing uses and lack of prioritization of outputs.	Ensure outputs of data are intuitive, easy to navigate and improved upon with feedback from users over time.
Reaching decision-makers	Using data in practice due to time constraints, users’ uncertainty of interpretation and lack of familiarity with tools.	Provide hands-on coaching; embed use within quality management cycles; engage improvement facilitators for change management support.

*Abbreviations*: *AFHTO* Association of Family Health Teams, *The Alliance* The Alliance for Healthier Communities, *BIRT* Business Intelligence Reporting Tool, *CHCs* community health centres, *CPCSSN* Canadian Primary Care Sentinel Surveillance Network, *D2D Data2Decisions*, *FHTs* family health teams, *HDC Discover* Health Data Coalition Discover, *Manitoba PCQI* Manitoba Primary Care Quality Indicators, *QIDSS* Quality Improvement Decision Support Specialists.

## Discussion

With this study, we set out to explore the current and potential use of EMR data for PHC performance measurement in the Canadian context. We aimed to capture the state-of-the-art of EMR data use as well as to gain practical insights for furthering its potential. To do so, we consulted both the literature and firsthand insights of system leaders, clinicians and researchers. We observe the following main findings.

First, while jurisdictions remain at varied stages [16], recognition of the importance and potential secondary uses of EMR data is common. Nonetheless, while nearly 15 years since the initial launch of a pan-Canadian PHC indicator set and almost a decade since its updating to include EMRs as a possible source, EMR data is used in only a handful of initiatives for performance measurement. Instead, a number of other data sources for PHC performance measurement continue to be relied on. This is predominately physician billing or other administrative sources such as census, laboratory and registry data and survey data. This finding is in line with recent international studies, signalling electronic health systems are yet to be leveraged to their full potential [14, 57]. These sources are in use for macro-level measurement across jurisdictions, be it in ad hoc reports, programme-specific monitoring and annual health system performance measurement, and at the micro-level as panel reports like in Alberta, Ontario, and Saskatchewan. It means, EMR data as a source for performance measurement is only a fraction of the total activity.

Where EMR data is in use, this is predominately geared towards performance measurement in the context of the micro-level, for use by individual clinicians and their teams. The EMR-based initiatives also equip affiliated physicians, their practices and networks with comparable data to generate research. EMR data for executives to manage and improve organizations is less established, though its potential is demonstrated by *BIRT* and *D2D*. Uses of EMR data for system performance improvement are not yet leveraged. This is despite its advantages, especially when linked with other data sets, to assess performance, identify problems such as unwarranted variation, and enable smarter resource allocation [14, 58]. Further to diversifying the performance measurement uses of EMR data, we note patients and the public are not among EMR data users at present, as the reporting across initiatives is not publicly available, nor is consistent patient access to their EMRs common practice.

The six different initiatives making use of EMR data for measurement and improvement demonstrate there is not a singular approach to do so. The initiatives vary in their contexts, including the target PHC practice model and affiliated EMR vendors, but also in their approaches to extract, standardize and return analyzed information to their users. In terms of the EMR-sourced indicators by each initiative, the range of indicators extend beyond the original 2012 pan-Canadian indicator set [38, 39], in particular with regards to chronic disease management and prescribing. Ways to update and broaden a pan-Canadian set of indicators that can potentially be sourced from EMR data should be explored together with continued investment in minimum data standards.

New initiatives in the past five years like *HDC Discover* and *Insights4Care*, as well as greater EMR coverage across jurisdictions, suggest the possibility for a quickening pace of change. The pan-Canadian nature of EMR vendors may facilitate the adoption of existing tools in other jurisdictions. Moreover, the COVID-19 pandemic has underscored the importance of timely, aggregated data for the system to monitor cases [40] as well as the potential use of EMR data in PHC to observe sudden changes in visits and to proactively reach patients [59].

To dramatically accelerate the use of EMR data will require more assertive action. The lessons for enabling EMR data use described by initiatives attest to the valuable experience and expertise that lies within the system and can be leveraged (Table 2), like advancing privacy and data sharing agreements.

The recurrent themes call for: defining a clear vision together with key stakeholders and focusing on the standardization of EMR data at the pan-Canadian level, as has been underscored elsewhere [15, 35, 60–62]; advancing beyond EMR adoption where still needed and investing in workforce competencies at all levels for the professionalization of performance measurement; and, considering updating the core set of pan-Canadian PHC indicators to fully account for the potential of EMR data as a source. Further research should test empirically the impact of EMR data for different decision-making uses. The implementation of EMR-sourced performance measurement and quality improvement should also leverage the insights of relevant international examples like the United Kingdom [63] and the Netherlands [64]. In particular, the further exchange of good practices around the handling of privacy and data sharing agreements and data capture in EMRs of virtual care services, mental health and addiction encounters, and socioeconomic status, appear needed.

### Strengths and limitations

To our knowledge, this is the first study to systematically explore and describe examples of EMR data use for performance measurement in the Canadian context from a health care performance intelligence perspective. The study was enriched by the wide-reaching engagement of experts across Canadian jurisdictions and of different profiles (stakeholders and clinician/researchers). Additionally, given the acceleration of electronic health information system improvements brought on by the COVID-19 pandemic, our findings are of particular relevance to ensure sustained, system-wide improvements are pursued.

Findings of this study should be understood in the context of three primary limitations. First, the target diversity in perspectives of informants was not met in all jurisdictions. While significant efforts were made for consistency in representation, the availability of informants, range of stakeholders and presence of research networks ultimately varies considerably by jurisdiction. The impact of this limitation was mitigated through the triangulation of existing sources and expert advice. Second, the process of classifying indicators involved a degree of subjectivity as our definition was broad and for this reason, we limited comparisons to indicator titles. Third, the analysis of key considerations was conducted by independent thematic coding. To limit the risk of overlooked considerations while also mindful of the burden the COVID-19 pandemic has placed on informants, a subset of the original informants reviewed these results.

## Conclusion

Performance measurement is integral to PHC improvement. In this study, we explored the use of EMR data for measurement and improvement in the Canadian context. As an evolving field, with continuous improvements in the maturity of EMRs across the country, we engaged informants of varied perspectives to systematically explore the extent of current use but also the potential use based on firsthand insights and experiences. The six initiatives identified, in general, share a common focus on practice, micro-level performance measurement and improvement. They also provide a range of insights into approaches to extract and display data, as well as the types of indicators analyzed using EMR data at present. These firsthand experiences, coupled with the momentum for digitalization in PHC brought on by 2020, should be leveraged to avoid unnecessarily slow progress and ensure the potential uses of EMR data across Canada and beyond, are realized

## Supplementary Information

**Additional file 1: Supplementary file 1.** Mapping of PHC actors. **Supplementary file 2.** Expert interview guide. **Supplementary file 3.** Characteristics of experts consulted. **Supplementary file 4.** Detailed mapping of indicator themes by initiative. 

## Data Availability

The datasets used and/or analysed during the current study are available from the corresponding author on reasonable request.

## Abbreviations

AFHTO: Association of Family Health Teams of Ontario
BIRT: Business Intelligence Reporting Tool
CPCSSN: Canadian Primary Care Sentinel Surveillance Network
D2D: Data2Decisions
EMRs: Electronic Medical Records
HDC: Health Data Coalition
PHC: Primary health care
