# Supplementary material for: The current and potential uses of Electronic Medical Record (EMR) data for primary health care performance measurement in the Canadian context: a qualitative analysis
Source: BMC Health Serv Res. 2021 Aug 15;21:820. doi: 10.1186/s12913-021-06851-0 (PMC8364440; doi:10.1186/s12913-021-06851-0)
Supplement: Supplementary file 1 — Additional file 1: Supplementary file 1. Mapping of PHC actors. Supplementary file 2. Expert interview guide. Supplementary file 3. Characteristics of experts consulted. Supplementary file 4. Detailed mapping of indicator themes by initiative. [file 12913_2021_6851_MOESM1_ESM.docx]

**The current and potential uses of Electronic Medical Record (EMR) data for primary health care performance measurement in the Canadian context: a qualitative analysis**

**Online Supplementary files**

**Supplementary file 1: Mapping of PHC actors**

| **Jurisdiction** | **Type** | **Actor (abbreviated name)^*^** |
| --- | --- | --- |
| **Newfoundland and Labrador** | Policy/ stakeholders | Newfoundland & Labrador Department of Health and Community Services^*^ |
|  |  | Eastern Health^*^ |
|  |  | eDocsNL |
|  | Research networks | Primary Healthcare Research and Integration to Improve Health System Efficiency (PRIIME)^*^ |
|  |  | Atlantic Practice Based Research Network (APBRN) |
| **Prince Edward Island** | Policy/ stakeholders | Health PEI^*^ |
|  | Research networks | Maritime Family Practice Research Network (MaRNet) |
|  |  | The Prince Edward Island Primary and Integrated Health Care Innovation Network (PIHcIN) |
| **Nova Scotia** | Policy/ stakeholders | Department of Health and Wellness^*^ |
|  |  | Nova Scotia Health Authority^*^ |
|  | Research networks | Nova Scotia Primary and Integrated Health Care Innovations Network |
|  |  | Maritime Family Practice Research Network (MaRNet) |
| **New Brunswick** | Policy/ stakeholders | New Brunswick Department of Health^*^ |
|  |  | New Brunswick Health Council |
|  | Research networks | New Brunswick SPOR Network: Primary and Integrated Community Care |
|  |  | Maritime Family Practice Research Network (MaRNet) |
| **Quebec** | Policy/ stakeholders | Ministère de la Santé et des Services sociaux – Ministry of Health and Social Services^*^ |
|  |  | Institut national d'excellence en santé et en services sociaux (INESSS) ­– National Institute of Excellence in Health and Social Services^*^ |
|  | Research networks | Reseau de recherche en soins primaires de l'Université de Montréal (RRSPUM) – University of Montreal Primary Care Research Network |
|  |  | Réseau-1 Québec – Quebec Knowledge Network in Integrated Primary Health Care |
|  |  | Pulsar^*^ |
|  |  | Groupe de recherche interdisciplinaire en informatique de la santé (GRIIS) – Interdisciplinary Research Group in Health Informatics^*^ |
| **Ontario** | Policy/ stakeholders | Ministry of Health and Long-Term Care |
|  |  | Ontario Health (Health Quality Ontario)^*^ |
|  |  | Alliance for Healthier Communities (the Alliance)^*^ |
|  |  | Association of Family Health Teams of Ontario (AFHTO)^*^ |
|  |  | OntarioMD^*^ |
|  |  | Institute for Clinical Excellence (ICES)^*^ |
|  | Research networks | eHealth Centre for Excellence |
|  |  | University of Toronto Practice Based Research Network (UTOPIAN)^*^ |
|  |  | Deliver Primary Healthcare Information Project (DELPHI)^*^ |
|  |  | The Eastern Ontario Network Practice-Based Research Network (EON) |
|  |  | McMaster University Sentinel and Information Collaborative (MUSIC) |
|  |  | Better Access and Care for Complex Needs (BEACCON) |
| **Manitoba** | Policy/ stakeholders | Manitoba Health, Seniors and Active Living^*^ |
|  |  | Shared Health Manitoba^*^ |
|  | Research networks | Manitoba Primary Care Research Network (MaPCReN)* |
|  |  | The Manitoba SPOR Primary and Integrated Health Care Innovation Network (MPN) |
|  |  | Manitoba Centre for Health Policy^*^ |
| **Saskatchewan** | Policy/ stakeholders | Ministry of Health^*^ |
|  |  | Health Quality Council of Saskatchewan (HQC)^*^ |
|  |  | Saskatchewan Medical Association (SMA) |
|  |  | eHealth Saskatchewan |
| **Alberta** | Policy/  stakeholders | Alberta Health^*^ |
|  |  | Alberta Health Services^*^ |
|  |  | Health Quality Council of Alberta (HQCA)^*^ |
|  |  | Alberta Netcare |
|  |  | Physician Learning Program |
|  |  | Alberta Medical Association (AMA) |
|  | Research networks | Southern Alberta Primary Care Research Network (SAPCReN)^*^ |
|  |  | Northern Alberta Primary Care Research Network (AFPRN) |
|  |  | The Alberta SPOR Primary and Integrated Health Care Innovation Network |
| **British Columbia** | Policy/  stakeholders | Ministry of Health |
|  |  | Provincial Health Services Authority (PHSA) |
|  |  | Doctors of BC |
|  |  | Health Data Coalition (HDC)^*^ |
|  |  | Northern Health |
|  |  | General Practice Services Committee (GPSC) |
|  |  | BC Patient Safety and Quality Council |
|  | Research networks | British Columbia Primary Care Research Network (BC-PCReN), University of British Columbia (BC-CPCSSN and SPOR PIHCIN)^*^ |
|  |  | eHealth Observatory, University of Victoria |
| **Yukon** | Policy/  Stakeholders | Yukon Health and Social Services |
|  |  | 1Health |
| **Northwest Territories** | Policy/ stakeholders | Department of Health and Social Services |
|  |  | Northwest Territories Health and Social Services Authority |
|  | Research networks | Institute for Circumpolar Health Research^*^ |
|  |  | Development of a Northern-Based SPOR Network in Primary and Integrated Health Care Innovations |
| **Nunavut** | Policy/  Stakeholders | Nunavut Department of Health^*^ |
|  | Research networks | Qaujigiartiit Health Research Centre^*^ |
| **Pan-Canadian** | Policy/ stakeholders | Health Canada |
|  |  | Canadian Institute for Health Information^*^ |
|  |  | College of Family Physicians of Canada |
|  |  | Canada Health Infoway |
|  |  | Canadian Medical Association |
|  |  | Canadian Partnership Against Cancer^*^ |
|  |  | Digital Health Canada |
|  |  | Canadian Nurses Association |
|  | Research networks | Canadian Primary Care Sentinel Surveillance Network (CPCSSN)^*^ |
|  |  | Strategy for Patient Oriented Research (SPOR) Primary and Integrated Health Care Innovations Network (PIHCIN)^*^ |

^*^Denotes actors from which an informant was directly engaged.

**Supplementary file 2: Expert interview guide**

**Information letter (adapted to exclude identifiers)**

**Background to the study**

Primary health care (PHC) and its strengthening through performance measurement is essential for sustainably working towards improved health outcomes. In Canada, a core set of PHC indicators with this aim and for use at the system- and practice-level across jurisdictions was first introduced in 2006 and has continued to be refined over time.

Since the initial release of PHC indicators for pan-Canadian use, the PHC Electronic Health Record (EMR) system has been recognized as an important data source. However, realizing the full potential of EMR data has been constrained by factors such as the quality and utility of largely unstructured data as well as the varied uptake of EMR systems across the country. As the adoption and sophistication of EMR systems advances, the extent to which PHC indicators are or could be sourced from EMR data has also improved.

While illustrative examples of EMR data use for performance measurement are known – such as in British Columbia, Manitoba and Ontario – an up-to-date overview of PHC indicators in use is needed as this context continues to evolve. An overview on the use and sourcing of PHC indicators is a relevant input for the continued updating of a pan-Canadian set. Moreover, exploring the contextual factors that influence the use of EMR data in the cases identified may offer insights into enabling system conditions and organizational factors.

**Aim of study**

This study aims to investigate the use of PHC indicators sourced from EMR data across jurisdictions in Canada in order to explore the current and potential use of EMR data and enabling contextual factors.

**Scope and guiding questions**

To scope our investigation, we have put focus on the use of PHC indicators drawing from routine administrative data or EMR systems. *Indicators* are defined as a quantitative measure that provides information about a performance dimension (e.g. quality, effectiveness, safety, people-centredness, etc.) of PHC. *Use* is defined as the selection, sourcing, analysis and dissemination of indicators for the purpose of performance measurement at the macro (policy), meso (organization/institution or network) or micro (practice) level. The study is guided by the following key questions.

1. **Reviewing the current use of EMR and/or other routine data sources such as administrative data for PHC indicators across jurisdictions**. Where is PHC EMR data or other routine data sources such as administrative data currently used as a source for PHC performance indicators across Canadian jurisdictions?
2. **Exploring the technical specifications of EMR-sourced indicators and context of their use**. Where PHC EMR data are in use, how are the indicators defined and standardized? How can the use of these indicators be further described with regards to their analysis, display and delivery to the target end-user?
3. **Analyzing the organizational factors enabling the use of EMR data for PHC indicators**. Where PHC EMR data are in use, how can the development of the EMR data system be described? What is the organizational context and model of primary care in which the data is being used?

**Approach to study**

The study is designed around two phases. The first sets out to identify where EMR data or other routine administrative sources are in use for PHC performance measurement at the system (macro), network (meso) or practice (micro) level across Canadian jurisdictions. This phase builds upon recent internal scans conducted by the Primary Health Care Information team of the Canadian Institute for Health Information (CIHI) and will be conducted through a systematic review of organizational websites. A series of key informant and stakeholder interviews will also be organized to gain further insights into ongoing activities across jurisdictions.

A second phase aims to explore in-depth the location-specific uses of EMR-sourced indicators. In this phase, the specific purposes of use and technical specifications for indicators will be studied through semi-structured interviews and document reviews. The context will also be explored with regards to the organization of the EMR data system and model of PHC in place.

**Dissemination and policy implications of results**

It is the intention of the study team to submit the findings for peer-reviewed publication. Key Informants will be noted in the acknowledgements unless requested otherwise. The study’s findings are expected to offer insights for the continued updating of a pan-Canadian set of PHC indicators by way of signalling high-frequency indicators or themes in use. Moreover, as the study endeavours to explore contextual factors that influence the use of PHC EMR data, the findings may offer insights into the EMR data system and organization of PHC enabling the uptake and use of EMR data. These findings may carry international relevance, offering insights to the strategic development of PHC and information systems that are also in alignment with a country’s model of care and the information needs of practitioners, managers and system decision-makers.

**Key considerations**

- Does your organization currently use EMR data or routine administrative data sources for PHC performance measurement? Is this defined in a framework or indicator set? Which PHC indicators does this include?
- What is the intended purpose of the indicator? (e.g. system planning, practice-level performance improvement)? How is the data being analyzed and disseminated? Who is the target end-user?
- Are you aware of any existing studies or reports or can suggest contacts that should be consulted in the scope of this work?

**Detailed semi-structured interview questions**

**1. Purposes of use of PHC performance indicators** (all experts)

- **Current uses**. How does your organization currently use PHC performance indicators? E.g. research, micro-level clinical practice improvement, meso-level organization or network planning, macro-level system monitoring?
- **Sources**. What are the current sources of data for the uses described? Specifically, which if any are sourced from EMR data?
- **Actors**. What actors do you work with directly in the scope of measuring PHC performance?
- **Planned uses**. What, if any, initiatives are currently in development using EMR data as a source for performance measurement?

**2. Description of current EMR-data uses** (only where applicable)

- **Overview of use**. Can you briefly describe the development of your organization’s current EMR data use overtime in terms of main milestones? How would you describe your current stage of use? Who are the target end-users (e.g. type of practices, physicians, total range of users engaged)?
- **Indicators**. What are the specific indicator themes being reported on? Are the technical specifications defined and if so, can these be shared? How have these changed overtime?
- **Data sources**. How is the EMR data being extracted? How does this extraction work across vendors? At what time-interval is data extracted? Where is the data stored? Is data anonymized?
- **Analysis**. How is the data currently analyzed (e.g. benchmarking, time trends, composite measures)? What comparators are used?
- **Display**. How is the data disseminated? What is the format of reporting (print, electronic, web-based)? What is the lag time if any in presenting analyzed data?
- **Delivery**. How do the findings reach the intended user? What is the reporting cycle? What resources are available to support end-users?
- **Impact evaluation**. Have studies on use and impact been conducted? What are the key findings where available? If anecdotally available, what are the lessons learned?

**3. Barriers or enablers of use** (all experts)

- **Opportunities for improvement**. In your opinion, how can your current uses of EMR data for performance measurement be improved upon?
- **Barriers**. In your opinion, what are the main obstacles or challenges faced by your organization or jurisdiction-at-large to make EMR data more actionable? In general, what are the obstacles to optimizing the use of EMR data for PHC performance measurement?
- **Enablers**. In your opinion, what are some of the factors that have contributed to achievements to-date?

**Other: Relevant reporting and additional informants**

- **Available reporting**. Are you aware of any existing studies or reports that are publicly available and/or can be shared in the scope of this work?
- **Additional informants**. Can you suggest a colleague, expert in your jurisdiction (specific stakeholder or clinician/research profile as needed) or network that you think should be met with in the scope of this work?

**Supplementary file 3: Characteristics of experts consulted**

| **Characteristics** | **Perspective** | | **Total informants n=61 (n=44^a^)** | |
| --- | --- | --- | --- | --- |
|  | **Policy-maker/**  **stakeholder** | **Researcher/**  **clinician** | **n**  (sub-total) | **%**  (total informants) |
| **Jurisdictions** | | | | |
| Newfoundland and Labrador | 3 | 1 | 4 | 7 |
| Prince Edward Island | 1 | 0 | 1 | 2 |
| Nova Scotia | 2 | 0 | 2 | 3 |
| New Brunswick | 4 | 0 | 5 | 8 |
| Quebec | 3^*^ | 2 | 5 | 8 |
| Ontario | 7 | 8 | 15 | 25 |
| Manitoba | 3 | 1 | 4 | 7 |
| Saskatchewan | 5 | 0 | 5 | 8 |
| Alberta | 5 | 2^*^ | 7 | 11 |
| British Columbia | 1 | 1 | 2 | 3 |
| Yukon | 0 | 0 | 0 | 0 |
| Northwest Territories | 0 | 2 | 3 | 5 |
| Nunavut | 1 | 1^*^ | 2 | 3 |
| Pan-Canadian | 6 | 2 | 8 | 13 |
| **Gender** | | | | |
| Female | 24 | 8 | 32 | 52 |
| Male | 17 | 12 | 29 | 48 |
| **Perspectives** | | | | |
| Policy-maker/stakeholder | - | - | 41 | 67 |
| Researcher/clinician | - | - | 20 | 33 |

Notes: ^a^In total 44 meetings were held. Nine interviews were conducted with two-person (or more) are counted as only one participant (meeting). ^*^ indicates correspondence in written format only (n=9).

**Supplementary file 4: Detailed mapping of indicator themes by initiative**

| **Indicator themes** | **Initiatives/organizations** | | | | | | |
| --- | --- | --- | --- | --- | --- | --- | --- |
|  | ***CIHI*** | ***CPCSSN*** | ***HDC Discover*** | ***Manitoba PCQI*** | ***Insights***  ***4Care*** | ***D2D*** | ***BIRT*** |
| Total indicators^a^ | 18 | NA**^b^** | 184 | 44 | 64 | 17 | 40+ |
| **Type** | | | | | | | |
| Process | 16 |  | 162 | 44 | 57 | 14 | 39 |
| Outcome | 2 |  | 22 | ­– | 7 | 3 | 3 |
| **Scope** | | | | | | | |
| Generic | 10 |  | 115 | 15 | 41 | 11 | 34 |
| Disease-specific | 8 |  | 31 | 29 | 23 | 6 | 8 |
| **Function** | | | | | | | |
| Prevention/screening | 10 |  | 29 | 16 | 16 | 5 | 6 |
| Prescribing | 3 |  | 62 | – | 18 | 1 | 4 |
| Disease management | 6 |  | 33 | 28 | 16 | 6 | 5 |
| Surveillance | – |  | 22 | – | 4 | ­– | 1+ |
| Other | – |  | 38 | – | 10 | 5 | 26 |

| **Prevention/screening** |
| --- |

| Well-baby | 1 | – | – | – | 1 | ­– | – |
| --- | --- | --- | --- | --- | --- | --- | --- |
| Blood pressure/CVD | 1 |  | 1 | 3 | 1 | 1 | 1 |
| Cancer screening | 3 |  | 3 | 3 | 6 | – | 3 |
| Other screening | ­– |  | 9 | 4 | – | 1 | 6 |
| Immunizations | 3 |  | 2 | 3 | 6 | 2 | 1 |
| Overweight/obesity | 1 |  | 6 | 1 | 1 | – | 1 |
| Physical activity | – |  | 4 | 1 | – | – | 1 |
| Smoking | 1 |  | 4 | 1 | 1 | 1 | 1 |
| SES | – | – | – | – | – | – | 3 |

| **Chronic disease management** |
| --- |

| Asthma | ­– |  | 2 | 1 | – | – | 1 |
| --- | --- | --- | --- | --- | --- | --- | --- |
| AMI/stroke | 1 | – | – | – | – | 1 | – |
| CAD | 2 |  | – | 5 | 3 | – | 1 |
| CHF | – | – | 4 | 5 | – | – | 1 |
| COPD | – |  | 4 | 4 | 4 | – | – |
| Diabetes | 1 |  | 7 | 7 | 8 | 4 | 2 |
| Hypertension | 2 |  | 2 | 5 | 4 | – | 1 |
| Kidney-related diseases | – |  | 4 | – |  | – | – |
| Mental health | 2 | ­ | 2 | – | 1 | 3 | 1 |
| Neurological | – |  | – | – | – | 1 | – |
| Musculoskeletal | – |  | 1 | 1 | – | – | – |
| Other/multi-conditions | – |  | 7 | – | ­– | 1 | 1 |

| **Prescribing** |
| --- |

| Antibiotics | – |  | 22 | – | 1 | – | – |
| --- | --- | --- | --- | --- | --- | --- | --- |
| Opioids/pain relief | – |  | 21 | – | 5 | – | 3 |
| Psychiatric | – |  | 4 | – | 4 | – | – |
| Other medications | – |  | 11 | – | 7 | – | – |
| Polypharmacy | – |  | 4 | – | 1 | 1 | 1 |
| **Other** |  |  |  |  |  |  |  |
| Care delivery | – |  | – | – | 1 | 1 | 22 |
| Patient status | – |  | – | – | 1 | 2 | 3 |
| Care bonuses | – | – | – | – | 5 | – | – |
| Document management | – |  | 38 | ­– | 3 | 2 | – |

Notes: ^a^This total is representative of the EMR-sourced indicators in each set and therefore, not necessarily the total indicators. Importantly, many of these indicator sets continue to be iterated. The total and themes of indicators is according to the versions available on the date reviewed. Refer to references for specific version number and date.

^b^As a surveillance database, the shading refers to the data elements available in CPCSSN.

–none specified. CAD: coronary artery disease; CDM: chronic disease management; CHF: coronary heart failure; COPD: chronic obstructive pulmonary disease.

Sources: (Association of Family Health Teams of Ontario 2017; CIHI 2012; Health Data Coalition 2020; Manitoba Health 2019; OntarioMD 2020).
